# Evolving Paradigms in Acute Myeloid Leukemia: Personalized Approaches to Therapy Across Age and Risk Groups

**DOI:** 10.3390/cancers17172824

**Published:** 2025-08-28

**Authors:** Sumeet K. Yadav, Utsav Joshi, Guleid Hussein, Mohamed Warsame, Bolun Liu, Abhash Shrestha, Peter Krastev, Hariprasad Reddy Korsapati, Amrit Singh

**Affiliations:** 1 Hospital Internal Medicine, Mayo Clinic Health System, Mankato, MN 56001, USA; warsame.mohamed@mayo.edu (M.W.); liu.bolun@mayo.edu (B.L.); korsapati.hariprasad@mayo.edu (H.R.K.); 2 Hematology and Medical Oncology Fellowship Program, Moffitt Cancer Center, Tampa, FL 33612, USA; utsav.joshi@moffitt.org; 3 Kathmandu Medical College, Kathmandu University, Kathmandu 44600, Nepal; abhashshrestha1@gmail.com; 4 College of Medicine, Kansas City University, Kansas, MO 64106, USA; krastev.peter79@gmail.com; 5 Department of Hematology and Oncology, Mayo Clinic Health System, Mankato, MN 56001, USA; singh.amrit@mayo.edu

**Keywords:** acute myeloid leukemia, molecular profiling, measurable residual disease, FLT3 inhibitors, IDH inhibitors, Menin inhibitors, CPX-351

## Abstract

Acute myeloid leukemia (AML) is an aggressive hematologic malignancy characterized by the clonal proliferation of myeloid precursors and rapid progression. Historically consisting of intensive chemotherapy, AML management has evolved significantly due to advances in molecular diagnostics and risk stratification. This review discusses current therapeutic paradigms in AML, emphasizing the growing role of personalized medicine across age and risk groups. For younger, fit patients, intensive regimens such as the “7 + 3” protocol remain the standard, often enhanced by targeted agents like FMS-like tyrosine kinase 3 (FLT3) and IDH inhibitors. Older or unfit individuals benefit from low-intensity treatments such as hypomethylating agents combined with venetoclax, now considered a frontline standard of care. The use of liposomal chemotherapy (CPX-351), measurable residual disease (MRD) monitoring, and maintenance therapy further refine post-remission strategies. Emerging therapies, including menin inhibitors, antibody–drug conjugates, and immunotherapies like CAR-T cells and vaccines, offer additional options, especially in relapsed/refractory settings. This comprehensive review outlines the current landscape and future directions in AML therapy, emphasizing the transition toward individualized, mutation-driven treatment strategies.

## Simple Summary

Acute myeloid leukemia (AML) is a life-threatening cancer of the blood and bone marrow that progresses rapidly. In recent years, the treatment landscape for AML has shifted from a one-size-fits-all approach to a more personalized strategy. This shift is driven by advances in understanding the genetic and molecular features of the disease, allowing for more targeted and less toxic therapies. This review summarizes current treatment approaches for both younger and older adults with AML, including the use of intensive chemotherapy, targeted therapies, hypomethylating agents, and novel agents like venetoclax and menin inhibitors. It also discusses how patient age, genetic mutations, and risk profiles guide treatment decisions, and highlights promising new therapies and immunotherapies under development.

## 1. Introduction

Acute myeloid leukemia (AML) is a clonal hematopoietic malignancy characterized by the proliferation of myeloid progenitor cells, leading to bone marrow failure [1]. AML accounts for about 1% of all cancers and is one of the most common types of leukemia in adults [2]. There are approximately 474,519 new leukemia cases worldwide, with AML representing around 25% of adult leukemia cases, translating to roughly 118,000 new cases annually [2]. In the United States, approximately 20,000 new cases are diagnosed annually [2]. AML incidence increases with age, with a median age at diagnosis of around 68 years [2]. It has a historically poor prognosis, particularly in the elderly and those with adverse genetic features [1].

AML is often considered a medical emergency because of its rapid progression that can lead to life-threatening complications within days to weeks [3]. Due to the aggressive nature of AML, patients need to be urgently diagnosed and started on treatment. Diagnosis typically involves identifying blasts of myeloid lineage in the bone marrow or blood by histology, flow cytometry and immunohistochemistry. Since the 2022 WHO classification a blast count of 20% is not required for certain AML subtypes such as those with the AML-defining genetic alterations NPM1, CBF-AML, and others [4,5]. Advances in molecular diagnostics and risk stratification have transformed the understanding and management of AML, allowing for a more personalized approach to therapy [6,7]. Cytogenetic abnormalities and somatic mutations such as FMS-like tyrosine kinase 3 (FLT3), nucleophosmin 1 (NPM1), isocitrate dehydrogenase 1 and 2 (IDH1/2), TP53, t(8;21), inv(16), etc., have become essential tools not only in prognosis determination but also in therapeutic decision-making [4,7,8]. Historically, intensive chemotherapy, typically the “7 + 3” regimen, was the cornerstone of AML treatment. However, outcomes were suboptimal in many subgroups, particularly older or medically unfit patients [1]. Over the last decade, treatment paradigms have evolved rapidly with the development of targeted therapies, hypomethylating agents, and combination regimens such as venetoclax-based low-intensity therapy [9]. These newer strategies have demonstrated significant improvements in remission rates, survival, and quality of life, particularly in populations previously considered difficult to treat [7,10].

This review provides a comprehensive, up-to-date synthesis of the AML treatment landscape, integrating frontline therapies, targeted agents, vaccines, and emerging immunotherapies within a unified framework that considers patient age, functional status, comorbidities, molecular risk factors, and disease setting (newly diagnosed vs. relapsed/refractory).

## 2. Molecular Landscape and Risk Stratification

The treatment of AML is guided by the patient’s functional status and the cytogenetic and molecular profile of the disease. Recurrent genetic abnormalities form the basis for risk stratification, classifying patients into favorable-, intermediate-, or adverse-risk categories. For example, t(8;21) and inv(16) are associated with favorable outcomes, while abnormalities like monosomy 7 and complex karyotypes are indicative of poor prognosis [1,7]. Commonly altered genes, including FLT3, NPM1, IDH1, IDH2, and TP53, not only influence prognosis but also serve as therapeutic targets. In particular, mutations in FLT3 and IDH provide opportunities for the use of specific inhibitors that have become integral components of AML treatment. Over the past decade, advancements in targeted therapies and risk stratification tools have significantly transformed the management of AML. Treatment is now increasingly personalized, with therapeutic strategies tailored to each patient’s molecular profile and overall clinical condition [9].

## 3. Frontline Treatment Approaches

An intensive chemotherapy backbone continues to remain a cornerstone for the treatment of AML, especially in younger and fit patients [11]. In recent years, this landscape has expanded with the approval of several targeted therapies based on their genetic mutations, such as FLT3 and IDH mutations [12,13]. Low-intensity chemotherapy regimens, along with hypomethylating agents (HMAs) such as azacitidine or decitabine, combined with the BCL-2 inhibitor venetoclax, have also become the new standard of care, especially in older or unfit patients [14,15]. Additionally, maintenance therapy for those who are not candidates for allogeneic stem cell transplantation is also gaining popularity.

There are validated tools for frailty and comorbidity assessments commonly used nowadays to assess fitness and determine fit patients who can undergo aggressive chemotherapy. Such tools include the G-8 geriatric screening tool, the Hematopoietic Cell Transplant–Comorbidity Index (HCT-CI), the SIE/SIES/GITMO consensus criteria, also known as the Ferrara criteria, the Eastern Cooperative Oncology Group performance status (ECOG-PS), the Charlson Comorbidity Score, and albumin level [16,17,18].

**Figure 1:**
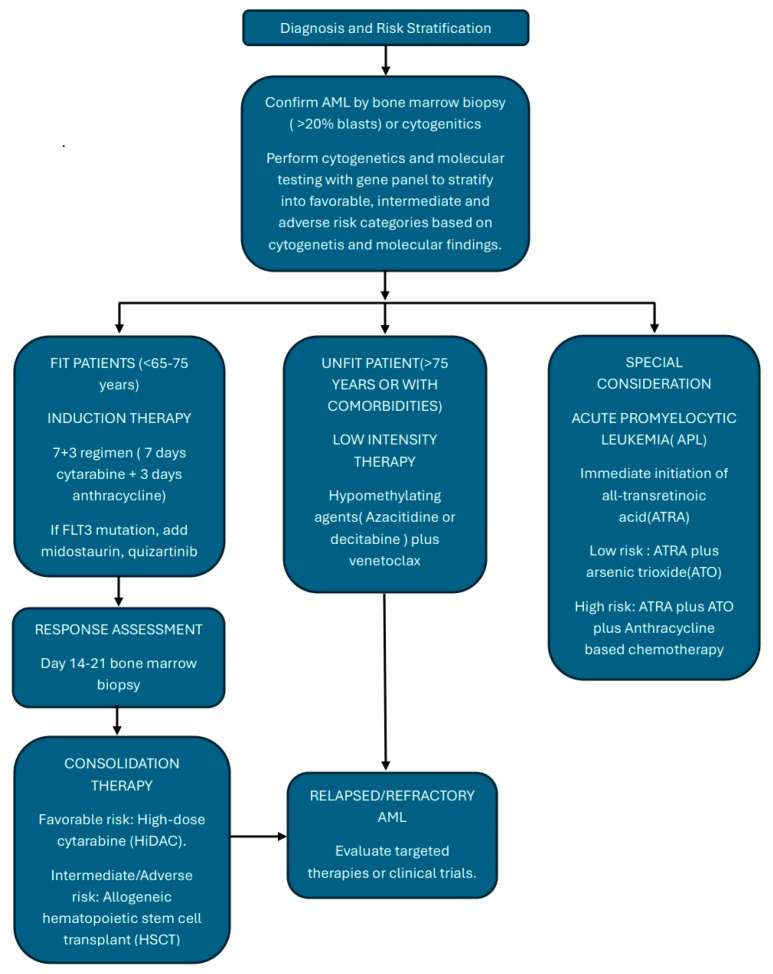
Overall pathway of diagnosis and treatment of AML.

In the following sections, we will discuss in detail treatment strategies based on the patient’s clinical profile. Figure 1 shows the overall pathway of diagnosis and treatment.

### 3.1. Young and Fit Patients

Patients under 60 years of age or 60 to 75 years of age without significant comorbidities and with a good functional status are usually considered for intensive chemotherapy. This is performed in two phases: Induction and consolidation with or without maintenance.

#### Induction and Consolidation Chemotherapy

Induction chemotherapy for AML patients includes the “7 + 3” regimen, which includes continuous infusion of cytarabine for 7 days, plus an anthracycline (daunorubicin or idarubicin) for 3 days [19]. This regimen has remained the standard of care since the 1970s, aiming to achieve complete remission (CR), which is defined as less than 5% blasts in the bone marrow and normalization of peripheral blood counts [19,20]. A dose-intensified version (daunorubicin 90 mg/m^2^ vs. 45 mg/m^2^) showed a superior outcome in younger adults (E 1900 trial). In this trial, 330 patients younger than 60 years were randomly assigned daunorubicin 90 mg/m^2^ vs. 45 mg/m^2^. Compared to the lower dose, the higher dose of daunorubicin resulted in superior overall median survival and complete remission rate [21]. However, there was no difference in complete remission between 60 mg/m^2^ and 90 mg/m^2^; rather, the higher dose was associated with increased mortality [22]. Patients with core-binding factor leukemias such as t(8;21) and inv(16) benefit from the addition of gemtuzumab ozogamicin (GO), as demonstrated in the ALFA-0701 and UK NCRI AML-17 trials [23]. A pivotal meta-analysis by Hill et al. assessed individual patient data from five randomized controlled trials evaluating the addition of GO to standard induction chemotherapy, which showed a reduced risk of relapse and improved overall survival. The 5-year OS improved from 39.7% with standard therapy to 44.3% with GO (HR 0.90; *p* = 0.01). The benefit was most pronounced in patients with favorable-risk cytogenetics, where the 5-year OS increased to 76.3% with the addition of GO [24].

Response to induction therapy is usually assessed with a day 14 bone marrow biopsy or a day 21 biopsy in case of the addition of FLT3 inhibitors. In cases with residual disease, a second induction cycle is considered [11]. If the complete response is not achieved after the second induction, the disease is deemed to be primary refractory.

We usually conduct consolidation in all cases and consolidation therapy depends on risk stratification. In favorable-risk AML, consolidation would be high-dose cytarabine (HiDAC) only for four cycles. For intermediate- and adverse-risk AML, allogeneic hematopoietic stem cell transplantation (HSCT) is recommended [25,26]. Since it takes time to arrange for BMT, in the interim, the patient receives consolidation with HiDAC. If FLT3 is positive, some may also use FLT3 inhibitors with HiDAC. Measurable residual disease (MRD) is used in a post-transplant setting to dictate maintenance therapy.

### 3.2. Older Patients

Older and frail patients with AML pose unique therapeutic challenges due to a higher prevalence of unfavorable cytogenetic or molecular risk profiles and complex medical comorbidities. A population study showed a 1-year survival rate of only ~2–30% in AML patients ≥60 years old, versus 50–70% in younger patients [27]. Over the last 10 years, treatment paradigms for newly diagnosed older or frail AML patients have evolved significantly, incorporating less intensive regimens and targeted agents (e.g., hypomethylating agents and bcl-2 inhibitor venetoclax).

#### 3.2.1. Induction and Consolidation with Conventional Chemotherapy in Older Patients

The overall survival in AML is strongly dependent on age and performance status. A real-world data analysis from the Swedish Acute Leukemia Registry on the decision to treat showed long-term survivors among the elderly who received intensive treatment despite their poor initial performance status [28]. Older patients may be considered fit for chemotherapy based on Eastern Cooperative Oncology Group performance status (ECOG-PS), comorbidity score, and frailty risk assessment [29,30].

Over the years, the treatment of AML in the elderly has shifted from traditional chemotherapy to personalized treatment and addition of targeted therapy. The combination of hypomethylating agents (HMAs) like azacitidine with the BCL-2 inhibitor venetoclax has emerged as a highly effective regimen and standard of care for older patients [15]. The phase III VIALE-A trial demonstrated that azacitidine combined with venetoclax significantly improved median OS compared to azacitidine alone (14.7 months vs. 9.6 months) and achieved higher complete response (CR) rates, 66.4% versus 28.3% [15]. Low-intensity regimens, including low-dose cytarabine (LDAC) + glasdegib, LDAC + venetoclax, HMA monotherapy (azacitidine or decitabine), andLDAC monotherapy, could also be used. Further supporting this low-intensity, targeted approach, studies combining HMA with IDH inhibitors have shown encouraging activity. The combination of ivosidenib and azacitidine yielded a CR rate of 78% and a median OS of 24 months in a phase 1b trial [31]. The phase III VIALE-C trial also showed similar median OS results for LDAC + venetoclax when compared to LDAC alone (8.4 months vs. 4.1 months) [32]. The BRIGHT AML 1003 phase II trial showed that a LDAC and glasdegib combination improved median OS compared to LDAC alone (8.8 months vs. 4.9 months) [33].

Fit older adults may still undergo intensive 7 + 3 chemotherapy, despite its utilization being very low in the older population. CR rates around 40–60% can be achieved, but early mortality is higher, remissions are less durable than in younger cohorts, and survival is not significantly longer compared to newer regimens [34,35].

Consolidation therapy in older patients is often attenuated due to excessive toxicity. HiDAC consolidation (standard in younger AML) is poorly tolerated in this group. Instead, older patients with favorable risk cytogenetics who underwent treatment with an intensive induction regimen and achieved remission CR may receive intermediate-dose cytarabine or a mini-consolidation regimen [36]. For fit older adults with intermediate risk cytogenetics who achieve remission, consolidation with allogenic transplant remains an option but the risk of non-relapse mortality remains high over 65 years [37,38]. HMA + venetoclax provides a gentler path to remission for frail and older patients (CR 36.7% in VIALE-A), with many achieving transfusion independence and bridging to transplant if their fitness improves [15]. Practical considerations with venetoclax regimens include prolonged myelosuppression, necessitating dose adjustments or therapy breaks, and the risk of tumor lysis syndrome in high-leukocyte AML.

#### 3.2.2. CPX-351 in Older Patients (Liposomal 7 + 3)

CPX-351 (vyxeos) is a liposomal formulation of cytarabine and daunorubicin in a fixed 5:1 molar ratio designed to enhance delivery to leukemia cells. CPX-351 was approved by the FDA and EMA to treat newly diagnosed secondary AML (sAML, including AML with myelodysplasia-related changes [AML-MRC] or therapy-related AML [t-AML]) in older adults above 60 years. In the pivotal Phase III trial for patients 60–75 years old with high-risk/sAML, CPX-351 demonstrated superior efficacy over conventional 7 + 3 chemotherapy with a median overall survival of 9.6 months vs. 5.9 months (HR 0.69, *p* = 0.003) [39]. Overall remission rates were also higher (47.7% vs. 33.3%) for the CPX-351 arm with a comparable safety profile to the 7 + 3 regimen. Notably, more patients (35% vs. 25%) were able to proceed to HSCT after remission in the CPX-351 arm, with sustained superior mOS after HSCT in this group (not reached vs. 10.25 months, HR 0.51). The 5-year overall survival was persistently higher in the CPX-351 arm (18% vs. 8%) [40]. A post hoc analysis further supports these findings across different European LeukemiaNet 2017 risk subgroups [41].

In a retrospective observational data analysis of AML patients who received CPX-351 (217 patients), patients who received Venetoclax and Azacitidine (439 patients) as the initial treatment showed similar overall outcomes; however, the Venetoclax and Azacitidine group demonstrated better tolerability, with fewer hospital admissions and infections [42].

## 4. Targeted Therapies in AML

**Figure 2:**
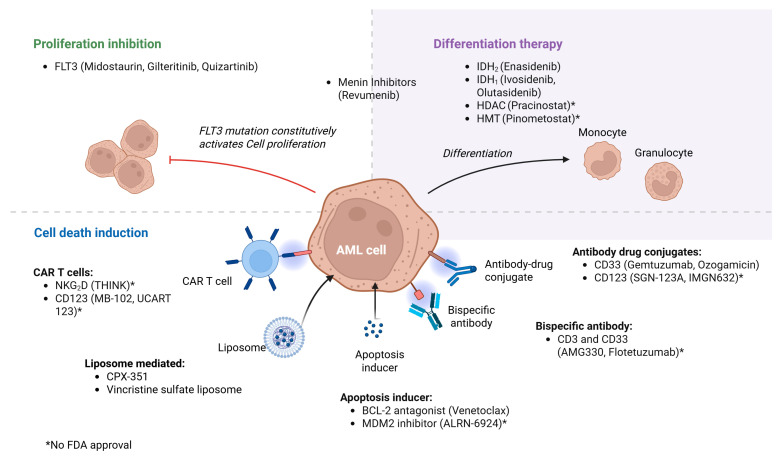
AML targets and therapies.

Molecular profiling of AML has enabled targeted therapies in AML patients and is used in patients with FLT3 and IDH1/2. They are often combined with chemotherapy in fit patients or HMA in unfit patients with great tolerability. These targeted drugs tend to be well tolerated, and common side effects are differentiation syndrome, indirect hyperbilirubinemia (with enasidenib), and cytopenias, but they lack the acute toxicities of chemotherapy. They are particularly relevant in older patients who may not tolerate intensive chemotherapy. The comparison of different targeted therapies is summarized in Table 1 and Figure 2.

### 4.1. FLT-3 Inhibition

FMS-like tyrosine kinase 3 (FLT3) mutations, particularly internal tandem duplications (FLT3-ITD), and tyrosine kinase domain (TKD) are among the most common molecular alterations in AML, occurring in approximately 25 to 30% of newly diagnosed patients. FLT3-ITD is a key prognostic marker, especially in patients with intermediate-risk cytogenetics, historically associated with increased relapse risk and poorer outcomes. While earlier guidelines emphasized the FLT3-ITD allelic ratio for risk stratification, the 2022 ELN update removed this parameter, reflecting evolving understanding and a lack of consistent predictive value. FLT3-TKD mutations currently do not affect ELN risk categorization, though they remain targetable with FLT3 inhibitors like midostaurin and gilteritinib.

The RATIFY (CALGB 10603) trial was a landmark study demonstrating that the addition of the multikinase inhibitor midostaurin to standard “7 + 3” induction chemotherapy significantly improved overall survival (74.7 vs. 25.6 months) in younger patients with FLT3-mutated AML, establishing midostaurin as a standard component of frontline therapy [12]. Other newer FLT3 inhibitors like gilteritinib, quizartinib, and crizotinib have been evaluated in older patients. Gilteritinib is FDA-approved in relapsed/refractory FLT3-mutated AML based on the ADMIRAL trial, which showed improved median OS vs. salvage chemotherapy (9.3 vs. 5.6 months, *p* < 0.001) [45]. A phase III LACEWING trial subsequently evaluated gilteritinib + azacitidine vs. azacitidine alone in newly diagnosed FLT3-mutated older AML, which found gilteritinib + azacitidine with a significantly higher CR rate (58.1% vs. 26.5%) but similar OS (9.8 months vs. 8.9 months) between the two groups. More recently, the QuANTUM-First trial evaluated quizartinib, a more selective and potent FLT3 inhibitor, in combination with intensive chemotherapy for FLT3-ITD AML. This study confirmed that quizartinib significantly prolonged overall survival compared to chemotherapy alone (31.9 vs. 15.1), further reinforcing the critical role of FLT3-targeted therapy in improving outcomes for this subset [43].

### 4.2. IDH1/2 Inhibitors

Mutations in isocitrate dehydrogenase 1 and 2 (IDH1/2) occur in approximately 15–20% of AML cases and contribute to leukemogenesis through the production of the oncometabolite 2-hydroxyglutarate, which blocks normal hematopoietic differentiation [7,51]. Two FDA-approved oral IDH inhibitors, ivosidenib (IDH1 inhibitor) and enasidenib (IDH2 inhibitor), have expanded treatment options for this subset [13,46]. The Phase III AGILE trial expanded the role of IDH-targeted therapy, demonstrating that the combination of azacitidine and ivosidenib significantly improved event-free and median overall survival compared to azacitidine alone in newly diagnosed patients with IDH1-mutant AML who were ineligible for intensive chemotherapy (24.0 months vs. 7.9 months) (12) [31]. These data support the incorporation of IDH inhibitors in frontline treatment regimens and suggest a paradigm shift toward mutation-directed therapy in AML, and it received FDA approval in 2022 for this indication.

In the relapsed/refractory setting, olutasidenib, a selective oral IDH1 inhibitor, has shown promising clinical activity. In a phase 1/2 trial, olutasidenib monotherapy-induced durable responses with an overall response rate around 35–40% in patients with relapsed/refractory IDH1-mutated AML, including some complete remissions, with a manageable safety profile [52]. Differentiation syndrome was reported but treatable, consistent with other IDH inhibitors. Olutasidenib adds to the growing armamentarium of mutation-targeted therapies in AML, offering an option for patients who have progressed on prior treatments.

Furthermore, the AG221-AML-005 phase 1b/2 trial compared enasidenib plus azacitidine versus azacitidine alone in patients with newly diagnosed IDH2-mutated AML who were ineligible for intensive chemotherapy [53]. The combination significantly improved the ORR (74% vs. 36%) compared with azacitidine alone. However, event-free survival results (15.9 months vs. 11.9 months, *p* = 0.11) and OS results (22.0 months vs. 22.3 months, *p* = 0.97) were not significantly different. Enasidenib monotherapy was also evaluated in a phase 3 trial for older patients with mutant IDH2 relapsed/refractory AML [54]. Enasidenib achieved better event-free survival (4.9 months vs. 2.6 months) compared with conventional salvage therapies, but failed to improve median OS (6.5 months vs. 6.2 months). Early dropout and the use of subsequent lines of therapies could have confounded these results.

### 4.3. BCL-2 Inhibition

The anti-apoptotic protein B-cell lymphoma 2 (BCL-2) is frequently overexpressed in AML, particularly in leukemic stem cells, contributing to resistance to conventional chemotherapy. Elevated BCL-2 expression in leukemic stem cells supports their survival and impedes apoptosis, thereby facilitating chemoresistance and disease persistence [55]. Targeting BCL-2 has emerged as a promising therapeutic strategy to overcome this resistance [56]. Venetoclax, a selective BCL-2 inhibitor, has transformed the treatment landscape for older or unfit patients with AML [57]. The phase III VIALE-A trial demonstrated that the combination of venetoclax and azacitidine significantly improved response rates and overall survival compared to azacitidine alone, with a composite complete remission (CR/CRi) rate of 66% and a median overall survival of 14.7 months [58]. Based on these results, venetoclax-based regimens have become a standard of care in this population. More recently, early-phase trials such as CAVEAT and V-FLAI have explored the use of venetoclax in combination with intensive chemotherapy in high-risk younger, fit adults with newly diagnosed AML. These studies have reported encouraging remission rates and manageable toxicity profiles, suggesting that venetoclax may have a broader role beyond the elderly population, potentially enhancing the depth and durability of responses in fit patients undergoing intensive induction [47,59]. Ongoing randomized studies will further clarify the optimal integration of venetoclax into the intensive regimen.

### 4.4. Menin Inhibitors—Beyond Single Agents

Menin is encoded by the MEN1 gene and is classically known as a tumor suppressor gene. In AML harboring rearrangements of mixed lineage leukemia (MLL), now also know as KMT2A, menin directly interacts with the KMT2A fusion protein to form a transcriptional complex which further drives aberrant gene expression (HOX, MEIS1, and PBX3) that blocks normal myeloid differentiation and promotes leukemic transformation [60,61]. Additional research also showed that NPM1 could be significantly affected by the menin-MLL regulation axis [62]. Menin inhibitors represent a novel targeted therapy class developed for these specific AML subsets with MLL rearrangements or NPM1 mutations, which constitutes a significant portion of AML patients with normal karyotypes [63].

In November 2024, the menin inhibitor revumenib was approved by the FDA for relapsed/refractory leukemia with KMT2A rearrangements. The pivotal phase I/II AUGMENT-101 trial investigated revumenib as a monotherapy in heavily pretreated patients with relapsed/refractory *KMT2Ar* AML [49]. The phase II part included 94 adult and pediatric patients with a median age of 37.0 years (range, 1.3–75.0), and 13 of them were above 65 years old. The overall response rate was 63.2% (95% CI, 49.3 to 75.6). In the efficacy-evaluable patients (*n* = 57), the CR + CRh rate was 22.8% (95% CI, 12.7 to 35.8) [49]. Another menin inhibitor, ziftomenib was evaluated in the phase I/II KOMET-001 trial, which also included older patients with relapsed or refractory AML. The trial reported an overall response rate (ORR) of 40% and a complete remission rate (CRc) of 35% in patients with NPM1 mutations [50].

More efforts have been implemented to investigate the efficacy of menin inhibitors in combination with other therapies, such as venetoclax and gilteritinib [64,65,66].

## 5. Post-Remission Therapy in Acute Myeloid Leukemia

Achieving CR in AML is essential but insufficient for long-term disease control. Despite CR, the majority of patients relapse due to persistence of MRD, especially in those with intermediate- or adverse-risk disease. There is also a considerable time gap between CR and transplant, which poses an increased risk. Therefore, post-remission therapy is a cornerstone of AML management aimed at eradicating residual leukemic cells and preventing relapse in the interim while awaiting HSCT [67].

In patients with favorable- or intermediate-risk cytogenetics, high-dose cytarabine (HiDAC) remains a standard consolidation regimen, particularly in younger, fit patients. A landmark CALGB study demonstrated that HiDAC significantly reduces relapse risk and improves disease-free survival compared to lower doses in patients under 60 years of age with core-binding factor AML [68]. Older patients or those with comorbidities may receive intermediate-dose cytarabine [22]. Patients who were induced with HMA/venetoclax (lower-intensity induction) may receive HMA only as a consolidation therapy, but they still possess a high risk of relapse [69].

Allo-HSCT remains the only curative option for intermediate- and high-risk AML or those with MRD positivity after induction/consolidation [70]. The European LeukemiaNet (ELN) recommends transplant for patients with adverse molecular/cytogenetic risk or persistent MRD, as post-transplant outcomes show significantly lower relapse rates and improved survival [4]. However, it is equally important to balance the benefits of allo-HSCT against the risk of transplant-related mortality and chronic graft-versus-host disease (GvHD) [71].

Maintenance strategies are increasingly used to prolong remission, especially for patients ineligible for allo-HSCT or with positive MRD post-transplant [72].The QUAZAR AML-001 trial showed that oral azacitidine significantly improved overall survival (24.7 vs. 14.8 months) and relapse-free survival (10.2 months vs. 4.8 months) in older patients in first remission who were not candidates for transplant [14]. This led to FDA approval of CC-486 for maintenance in AML in 2020. In FLT3-ITD-mutated AML, the SORMAIN trial demonstrated that sorafenib maintenance post-transplant significantly reduced relapse risk and improved relapse-free survival [73]. Other FLT3 inhibitors like midostaurin and gilteritinib are under evaluation in both transplant and non-transplant maintenance settings [72]. In the post-transplant setting, the MORPHO trial investigated gilteritinib, a FLT3 inhibitor, as maintenance therapy in patients with FLT3-ITD-mutated AML following allogeneic HSCT. While the trial did not achieve its primary endpoint of improved relapse-free survival in the overall population, a pre-specified subgroup analysis revealed that patients with detectable MRD before or after transplant experienced a significant benefit from gilteritinib maintenance, with a hazard ratio of 0.515 (95% CI, 0.316 to 0.838; *p* = 0.0065) [72]. While not yet approved for maintenance, studies are exploring ivosidenib (IDH1) and enasidenib (IDH2) to reduce relapse in patients with IDH-mutated AML post-remission [74]. Venetoclax combinations are under investigation in the maintenance setting. Given its synergy with hypomethylating agents and activity in MRD-positive disease, venetoclax is considered promising for both transplant-ineligible and post-transplant maintenance [75]. Emerging data support the use of menin inhibitors (e.g., revumenib, ziftomenib) in patients with KMT2A-rearranged or NPM1-mutated AML. Their role in maintenance is under investigation, particularly in MRD-positive cases after achieving remission with prior lines of therapy [76].

## 6. Measurable Residual Disease (MRD) and Treatment Monitoring

Measurable residual disease is a strong independent predictor of relapse and survival. Techniques such as multiparameter flow cytometry, PCR for NPM1 or fusion genes, and next-generation sequencing (NGS) allow detection of residual disease below morphologic thresholds [77]. MRD-positive patients may benefit from intensified consolidation, allo-HSCT, or inclusion in maintenance trials. The incorporation of MRD monitoring into treatment algorithms is now standard in many centers and trials [78].

## 7. Future Directions

The landscape of post-remission AML therapy is evolving toward precision-based, MRD-driven approaches. Combinations of novel agents—such as menin inhibitors, immune therapies (e.g., CD123 or CD47 antibodies), and vaccines—with or without chemotherapy are being evaluated in maintenance and consolidation settings. Personalized risk-adapted therapy, based on molecular profiling and MRD dynamics, is likely to define future standards for durable remission in AML.

### 7.1. Antibody–Drug Conjugates

Antibody–drug conjugates, or ADCs, comprise a monoclonal antibody conjugated to a cytotoxic drug. They allow for targeted cytotoxicity and death of leukemic cells with minimal off-target effects [79]. Gemtuzumab ozogamicin is a well-known ADC comprising a monoclonal antibody targeting the CD33 antigen and the cytotoxic agent calcheamicin [80]. Studies have shown improved survival in patients treated with gemtuzumab ozogamicin alone or in combination with other chemotherapeutic agents [79]. CD33 is a sialic acid-binding immunoglobulin-like lectin molecule that is expressed on the surface of myeloid progenitor cells amd differentiated myeloid cells, as well as normal hematopoietic stem cells. It is the expression of this antigen on normal hematopoietic stem cells that mediates its hematologic toxicity despite its demonstrated clinical efficacy [79,80]. In order to mitigate this limitation, alternate targets with more restricted expression patterns are under investigation, including LILRB4, CLL-1, and CD123 [81,82,83].

Lintuzumab-Ac225 (Actimab-A) is an anti-CD33 radioimmunoconjugate that delivers the alpha-emitting radionuclide Actinium-225 to leukemic cells, offering potent cytotoxicity with a different mechanism of action [84]. Another promising ADC is IMGN632 (Pivekimab sunirine), which targets CD123 and is conjugated to a DNA-alkylating agent, showing encouraging early clinical activity in relapsed or refractory AML and minimal toxicity to normal hematopoietic stem cells due to its more selective target profile [85,86].

### 7.2. Immunotherapy and Immune Checkpoint Inhibitors

Immune checkpoint inhibitors (ICIs) have been used in the treatment of AML with varied results. Monotherapy with ICIs have shown limited efficacy; however, their combination with HMAs has yielded improved response rates specifically in relapsed/refractory AML [87,88]. ICIs targeting Cytotoxic T-Lymphocyte Associated Protein (CTLA-4), Programmed Cell Death 1 (PD-1) and Programmed Cell Death-Ligand 1(PDL-1)—key regulators in immune tolerance—are currently studied extensively in clinical trials, but none showed a significant advantage of addition (12).

PDL-1 is expressed on the surface of AML cells where it engages the PD-1 receptor on T cells, inhibiting their function. CTLA-4 is expressed on T cells and binds CD-28, inhibiting T-cell activation. By binding these different targets, ICIs restore or enhance T-cell activation, potentially causing leukemic cell death [89]. Despite the theoretical appeal, clinical trials of immunotherapies in AML have yielded mixed results. For example, early studies evaluating immune checkpoint inhibitors targeting PD-1/PD-L1 pathways, such as nivolumab or pembrolizumab, have not demonstrated consistent improvements in overall survival when used as monotherapy or in combination with chemotherapy in AML patients [90,91]. Similarly, the addition of the anti-CD47 antibody magrolimab to standard therapy showed promising results in phase II but was terminated early in phase III due to inferiority to standard treatment [92].

### 7.3. Bi-Specific T-Cell Engagers (BiTEs) and Chimeric Antigen Receptor T-Cell (CAR-T) Therapy

Bi-specific Engagers (BiTEs) is an immunotherapeutic agent that integrates two binding domains within a single entity. AMG330 is one example—it binds CD33 on AML cells and CD3 on T cells, resulting in T-cell-mediated cytotoxicity [93]. Clinical trials investigating AMG330 as well as other BiTEs are currently underway. Notably, in a first-in-human, open label, dose-escalation study performed by Ravandi et al., 77 patients were treated with AMG330 on 14-day or 28-day cycles. Out of 60 patients who were able to be evaluated, 8 experienced complete remission or a morphologic leukemia-free state. Among the 52 that did not respond, 37% showed a decline of 50% or more in bone marrow percentage [93].

Chimeric Antigen Receptor T-cell Therapy (CAR-T) involves genetically engineered T cells with receptors specific to antigens expressed on the surface of the cancer cell. It is a treatment modality that has been successful in the treatment of lymphoid malignancies. CAR-T cells specific to CD33 and CD123 on AML cells have shown safety and feasibility in clinical trials for relapsed/refractory AML [87].

### 7.4. Vaccines and Dendritic Cell Therapies

AML vaccines have shown safety and efficacy in elderly patients. Vaccines targeting Wilms’ Tumor 1 (WT1) antigen, proteinase 3 and RHAMM antigens have been explored. Vaccines targeting WT-1 hold enormous potential accounting for its high expression and mutation rates in AML cells. They are believed to induce AML cell death by triggering a robust immune response [94].

Dendritic Cell-Based Vaccines or DC-based vaccines involve allogenic or autologous DCs primed with antigens specific to AML in order to stimulate an immune response. DCs are extremely effective antigen-presenting cells and play an important role in the activation of T cells. A systematic review by Khanmiri et al. assessed 11 trials and found that DC vaccine therapy was associated with a complete or partial remission in addition to stabilization of disease. They also reported an enhanced immune response as evidenced by the increased level of Th1 cytokines, CD4^+^ and CD8^+^ T cells, WT1-specific T cells, and activated natural killer (NK) cells [95].

While some of these results are encouraging, AML and DC-based vaccines remain emerging therapies and will need further dedicated research.

## 8. Conclusions

The management of Acute Myeloid Leukemia has undergone a remarkable transformation, driven by advances in molecular profiling, risk stratification, and the development of novel therapies. No longer a one-size-fits-all approach, AML treatment is now highly individualized, guided by patient fitness, genetic and cytogenetic profiles, and measurable residual disease. For younger, fit patients, intensive chemotherapy remains the backbone of treatment, often supplemented with targeted therapies like FLT3 or IDH inhibitors. For older or unfit patients, venetoclax, in combination with hypomethylating agents and other lower-intensity regimens, offers an effective alternative with reduced toxicity.

While challenges remain, particularly in relapsed/refractory AML and those with adverse-risk disease, the integration of targeted agents, improved supportive care, and expanding transplant eligibility are steadily improving outcomes. Ongoing clinical trials, development of immunotherapies, and real-time use of measurable residual disease to guide therapy promise further advances. Ultimately, the future of AML treatment lies in increasingly precise, biology-driven strategies that balance efficacy with tolerability across all patient populations.

## Figures and Tables

**Table 1:** Summary of targeted therapies in AML.

Target	Key Trial	Drug	Setting	Treatment	Complete Response	OS in Months (Median Followup)
FLT3	RATIFY [12]	Midostaurin	Newly diagnosed FLT3-mutant AML	Midostaurin + 7 + 3 vs. standard 7 + 3	59% vs. 54%	74.7 vs. 25.6; HR 0.78, *p* = 0.009 (59 Months)
FLT3	QuANTUM-First [43]	Quizartinib	Newly diagnosed FLT3-ITD AML	Quizartinib + 7 + 3 in induction and Quizartinib + HiDAC in consolidation vs. standard chemo	55% vs. 55%	31.9 vs. 15.1; HR 0.78, *p* = 0.03 (39.2 Months)
FLT3	QuANTUM-R [44]	Quizartinib	Relapsed/Refractory AML with FLT3-ITD	Quizartinib vs. salvage chemotherapy		6.2 vs. 4.7; HR 0.76, *p* = 0.02 (23.5 Months)
FLT3	ADMIRAL [45]	Gilteritinib	Relapsed/Refractory AML with FLT3-ITD or TKD	Gilteritinib vs. salvage chemo	34%	9.3 vs. 5.6; HR 0.64, *p* = 0.001 (17.8 Months)
IDH1	AGILE [31]	Ivosidenib	Newly diagnosed IDH1-mutant AML	Ivosidenib + azacitidine vs. azacitidine	47.2% vs. 14.9%	24.0 vs. 7.9; HR 0.44, *p* < 0.001 (12.4 months)
IDH1	AG120-001 [13]	Ivosidenib	Relapsed/Refractory IDH1-mut AML	Single-arm Ivosidenib	21.6%	8.8 (14.8 Months)
IDH2	AG221-001 [46]	Enasidenib	Relapsed/Refractory IDH2-mutant AML	Single-arm Enasidenib	19.3%	9.3 (10.9 Months)
BCL-2	VIALE-A [15]	Venetoclax	Older/unfit AML	Venetoclax + azacitidine vs. azacitidine	66.4% vs. 28.3%	14.7 vs. 9.6; HR 0.66, *p* < 0.001 (20.5 months)
BCL-2	VIALE-C [32]	Venetoclax	Older/unfit AML	Venetoclax + low-dose cytarabine (LDAC) vs. LDAC	48 % vs. 13%	8.4 months vs. 4.1; HR 0.70, *p* = 0.04 (17.5 months)
BCL-2	CAVEAT [47]	Venetoclax	Elderly fit AML	Single-arm Venetoclax + high-dose cytarabine (HiDAC) + idarubicin	75%	19.3 months (31.3 months)
BCL-2	M14-358 [48]	Venetoclax	Elderly Unfit AML	Single-arm Venetoclax with azacitidine or decita-bine	44% in venetoclax plus azacitidine and 55% in venetoclax plus decitabine	16.4 (29 months) in Azacitidine and 16.2 (40 months) months in decitabine
Menin (KMT2A/NPM1)	AUGMENT-101 [49]	Revumenib	Relapsed/Refractory KMT2A-rearranged AML or ALL	Single-arm Revumenib	23%	Not reported (early phase)
Menin (NPM1)	KOMET-001 [50]	Ziftomenib	Relapsed/Refractory NPM1-mutated AML	Single-arm Ziftomenib	25%	Not reported (early phase)
